# Early‐onset atopic dermatitis and food hypersensitivity increase the risk of atopic march

**DOI:** 10.1111/cea.14189

**Published:** 2022-06-28

**Authors:** Maria Pesonen, Markku J. T. Kallio, Annamari Ranki, Martti A. Siimes, Kirsi M. Järvinen

**Affiliations:** ^1^ Finnish Institute of Occupational Health Occupational Health Unit Helsinki Finland; ^2^ Hospital for Children and Adolescents, Helsinki University Hospital University of Helsinki Helsinki Finland; ^3^ Department of Dermatology and Allergology University of Helsinki and Helsinki University Hospital HUS Helsinki Finland; ^4^ Department of Pediatrics, Division of Allergy and Immunology & Center for Food Allergy University of Rochester Medical Center Rochester New York USA

**Keywords:** adolescents, allergic rhinoconjunctivitis, asthma, children, recurrent wheezing, sensitization, skin prick test

**Key messages**

- The atopic march occurred in 6% of healthy infants followed from birth to adulthood.
- Children with a combination of early‐onset AD and FHS might represent a high‐risk population.
- Minority (22%) of subjects with atopic disease and/or sensitization in childhood were symptom‐free in adulthood.

To the Editor,

The prevalence of the atopic march, described as a progression from atopic dermatitis (AD) early in life to allergic respiratory disease, is debated, and the relatively recently appreciated association of food allergy to atopic dermatitis calls for further studies to better define the role of food allergy in progression of atopic march. We had the opportunity to study the succession of atopic diseases in healthy newborns prospectively followed from birth until early adulthood. Our aim was to clarify how often infantile or childhood AD predicted subsequent recurrent wheezing (RW) and/or allergic rhinoconjunctivitis (ARC) and whether food hypersensitivity symptoms (FHS) played a role in the progression into atopic march, as suggested.^1^

We analysed data of a prospective 20‐year follow‐up study initiated in 1981 of 200 healthy newborns derived from the general population.^2^ Of the 200 infants, 84 (42%) had a family history of allergy, defined as having at least one first‐degree relative with allergic symptoms.

The infants visited the paediatric clinic and were clinically examined by a paediatrician at ages 2, 4, 6, 9, and 12 months. A total of 163 (82%) of the children were re‐assessed at age 5 years, 150 (76%) at 11 years, and 164 (83%) at 20 years with skin prick testing (SPT), clinical examination, and structured interviews by a study physician to record the presence of AD, recurrent wheezing (RW), allergic rhinoconjunctivitis (ARC) during the preceding year and allergic sensitization as assessed by SPT. At ages 5 and 11 years, parent‐reported food hypersensitivity symptoms (FHS, itching or swelling of the lips, oral mucosa or throat, urticarial eruption, or severe vomiting within 2 h of ingestion of a specific food) during the preceding year were recorded.

For the purposes of this study, early‐onset AD was defined as AD present at ≥2 follow‐up visits in the first year of life; late‐onset AD appeared by age 5 years. The atopic march was defined as a history of early‐onset or late‐onset AD and subsequent development of RW and/or ARC independent of their order of appearance. As there were no follow‐up visits between ages 1 and 5 years, the exact age of onset in late‐onset AD could not be reliably recorded.

For details of definitions of atopic diseases, SPTs, statistics and ethical considerations, see the Complementary file (DOI https://doi.org/10.5281/zenodo.6674756) at Zenodo (https://zenodo.org/).

The prevalence of atopic diseases at ages 5, 11 and 20 years and the demographic data of this cohort have been previously reported.^2^ Co‐morbidity of ARC, AD, and RW at ages 5, 11, and 20 years is presented in Figure 1. Prevalence of AD remained relatively stable during the follow‐up but peaked at 20 years (21%), as did RW (12%), ARC (42%) and allergic sensitization (52%, not shown in Figure 1), whereas the prevalence of ARC dramatically increased from age 5 to 20 years (from 4% to 42%), that of AD increased only slightly from 17% to 21% and RW from 6% to 11%. The difference between those with or without allergic family history was that AD was more prevalent in those with allergic family history at 5 and 20 years but not at 11 years, whereas ARC increased more rapidly in those with allergic family history throughout childhood, and more RW was seen in them only at 20 years.

**FIGURE 1:**
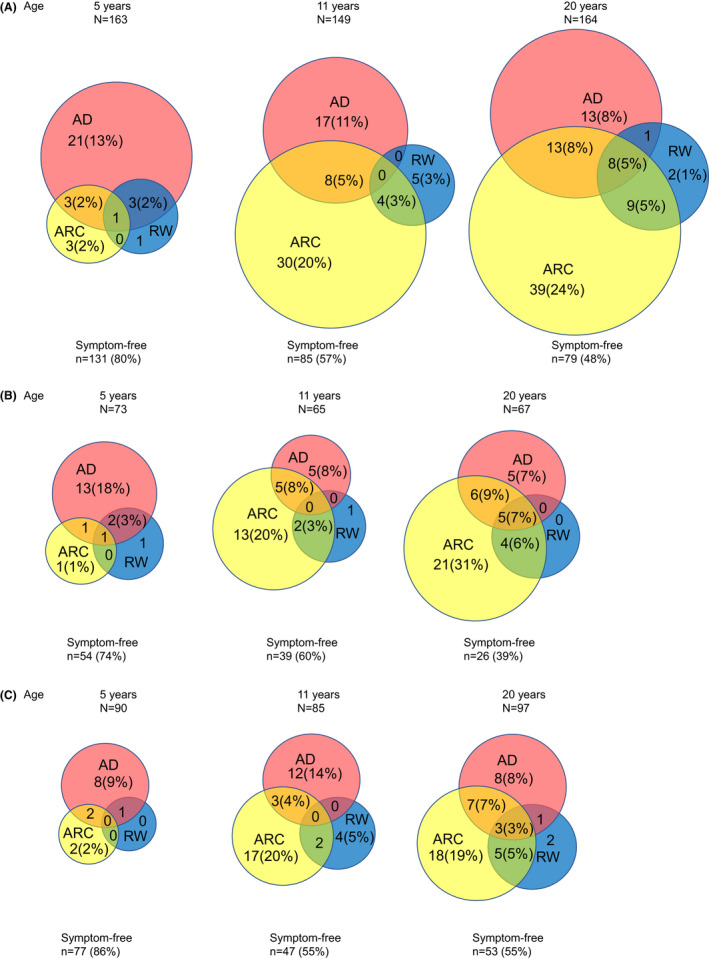
Prevalence and co‐morbidity of atopic dermatitis (AD), allergic rhinoconjunctivitis (ARC), and recurrent wheezing (RW) at ages 5, 11, and 20 years. A, all subjects; B, subjects with family history of allergy; C, subjects without family history of allergy. Percentages of the total of participants at each follow‐up in parentheses

Early‐onset AD was present in 8% and late‐onset AD in 17% of subjects. Of the 15 children with early‐onset AD, 50% also had AD at age 5 years, whereas 14% of the 183 children without early‐onset AD developed it by age 5 years (*p* = 0.001, Table 1). FHS at ages 5 and 11 years was more common in children with early‐onset AD than in those without (*p* = 0.01 and 0.01, respectively). Similarly, ARC was more common at ages 5 and 20 years (*p* = 0.02 and 0.01, respectively), and sensitization was more common at ages 5 and 11 years in those with early‐onset AD versus those without (*p* = 0.001 and *p* = 0.01, respectively). RW at age 20 years (*n* = 20) was more common in those with early‐onset AD (*p* = 0.01). Of children with FHS at age 5 years, 43% (6 out of 14) had developed RW by age of 20 years compared to 10% (12 out of 125) of children without FHS (*p* = 0.002). ARC at age 20 years was also more common in subjects with FHS at age 5 years than in those without FHS, 71% (10 out of 14) versus 39% (49 out of 125), *p* = 0.02.

**TABLE 1:** Association of AD during 1st year of life (early AD) and subsequent atopic diseases and sensitization by SPT positivity. A subject may have more than one atopic disease recorded at a given follow‐up visit

	Children (*n* = 15) with early AD, *n* (%)	Children (*n* = 183) without early AD, *n* (%)	P	OR	95% CI
Outcomes at age 5 years (*N* = 163)					
AD (at age 5 years)	7 (50)	20 (14)	0.001	6.44	1.8–22.8
FHS	5 (36)	14 (10)	0.01	5.25	1.5–18.9
RW	1 (7)	4 (3)	0.65 (NS)		
ARC	3 (21)	4 (3)	0.02	7.77	1.6–39.3
SPT positivity	7 (50)	17 (12)	0.001	8.07	2.3–28.1
Outcomes at age 11 years (*N* = 149)					
AD (at age 11 years)	3 (30)	22 (16)	0.27 (NS)		
FHS	4 (40)	12 (9)	0.01	6.87	1.5–30.7
RW	1 (10)	8 (6)	0.98 (NS)		
ARC	4 (40)	36 (26)	0.22 (NS)		
SPT positivity	8 (80)	49 (35)	0.01	8.71	1.7–43.9
Outcomes at age 20 years (*N* = 164)					
AD (at age 20 years)	6 (46)	28 (19)	0.02	4.1	1.2–14.7
RW	5 (38)	15 (10)	0.01	6.12	1.6–23.4
ARC	10 (77)	58 (39)	0.01	6.77	1.7–27.2
SPT positivity	10 (77)	75 (51)	0.06 (NS)		

*Note:* χ^2^ test, and in case of small numbers (≤5 subjects in a cell), Fisher's exact test were used to measure statistical significance. OR and CI calculated with multivariate logistic regression.

Abbreviations: AD, atopic dermatitis; ARC, allergic rhinoconjunctivitis; CI, confidence interval; NS, not significant; OR, odds ratio; RW, recurrent wheezing; SPT, skin prick test.

A total of 12 subjects (6% of the cohort) had a history of atopic march, defined as early or late‐onset AD that preceded the development of respiratory atopic disease (RW and/or ARC). Of the 15 children with early‐onset AD, six (40%) developed “atopic march after early‐onset AD”, that is, subsequent ARC (two cases) or both ARC and RW (four cases) by age 20 years. Of the 20 subjects who had late‐onset AD, 6 (30%) developed “atopic march after late‐onset AD”, that is, subsequent ARC (four cases) or RW (two cases) by age 20 years. Regarding FHS, one‐third (5 out of 14 present at 5‐year‐follow‐up visit, 36%, Table 1) of the children with early‐onset AD, 25% (5 out of 20) of those with late‐onset AD and 5% (7 out of 128) of those without AD had FHS at age 5 years.

Among the children with early‐onset AD, those with FHS at age 5 years (4 out of 5) were more likely to have RW at age 20 years than those without FHS (1 out of 8), *p* = 0.03. In fact, all the five subjects with the combination of early‐onset AD and FHS at age 5 years had respiratory atopic disease both in childhood (at age 5 or 11 years) and in adulthood and were SPT positive at ages 5, 11, and 20 years. In contrast, in children with late‐onset AD, the presence of FHS was not significantly associated with subsequent respiratory atopic disease.

In the subgroup of 118 subjects who participated all the follow‐up visits (during the first year of life and at ages 5, 11, and 20 years; complete follow‐up), we compared the prevalence of atopic diseases in young adulthood to the history of atopic manifestations in childhood. Most individuals with ARC in adulthood (88%) had a history of respiratory atopic disease and sensitization, but not AD, in childhood. On the contrary, all the subjects with RW at age 20 years had a history of atopic disease and/or sensitization in childhood, most of them (71%) having a history of childhood AD. In contrast, AD in adulthood was not significantly associated with childhood respiratory atopic diseases or sensitization and was not preceded by childhood AD in nearly half (48%) of the cases.

For more detailed results, additional tables and figures, see the Complementary file at Zenodo (https://zenodo.org/).

The atopic march may be relatively infrequent among unselected populations. In our cohort derived from the general population, the frequency of the atopic march was 6%, while in a previous study on two population‐based cohorts, it was estimated to 7%.^3^ However, the rates of the atopic march might be higher in high‐risk cohorts with atopic parents^4^ and, according to our study, in those with onset of AD in early childhood. In our cohort, subjects with the atopic march comprised 40% of the infants with early AD and 30% of those with late‐onset AD. Notably, in our cohort, none had a history of early AD and the development of first RW and then ARC, a pattern of the atopic march sometimes defined in literature.^3^ Interestingly, all the five children with the atopic march after early AD had developed FHS by age 5 years. The association of early AD and subsequent FHS is consistent with previous reports.^5^, ^6^, ^7^ As the number of these children is small in our cohort, and no firm conclusions on the role of FHS in the atopic march may be drawn based on the present study. Previous studies have confirmed the developmental sequence of atopic march with AD predisposing to food allergy and food allergy preceding airway disease.^4^ Sensitization to food allergens, therefore, appears to be significant in the progression of AD to RW. The inflamed skin along the disrupted barrier in AD might increase the risk of sensitization to food *via* the skin.^8^, ^9^ Contrasting to RW, ARC at age 20 years was not associated with childhood AD in our study, which might suggest a role for a disrupted barrier function specific to the development of RW.

Although the small numbers of subjects in the subgroups in our study does not allow drawing definitive conclusions, subjects with early‐onset AD and FHS might represent a high‐risk population for atopic march. Our findings from a clinical follow‐up study call for further investigation into the connection between AD, FHS, and asthma, which might have important implications for design of preventive measures against atopic diseases. For strengths, limitations, and additional discussion of the context, see the Complementary file at Zenodo (https://zenodo.org/).

## AUTHOR CONTRIBUTIONS

Study design: KMJ and MP. Data manipulation and analysis: MP and MJTK. Writing and/or reviewing manuscript: All authors. Approval of final manuscript: All authors.

## CONFLICT OF INTEREST

None to be declared by the authors. The sponsors of the study did not have any involvement in designing the study, in the collection, analysis and interpretation of data, writing of the report, and in the decision to submit the paper for publication.

## Data Availability

The data is not publicly available.

## References

[1] Davidson WF , Leung DYM , Beck LA , et al. Report from the National Institute of Allergy and Infectious Diseases workshop on "atopic dermatitis and the atopic march: mechanisms and interventions". J Allergy Clin Immunol. 2019;143:894‐913. 30639346 10.1016/j.jaci.2019.01.003 PMC6905466

[2] Pesonen M , Kallio MJ , Ranki A , Siimes MA . Prolonged exclusive breastfeeding is associated with increased atopic dermatitis: a prospective follow‐up study of unselected healthy newborns from birth to age 20 years. Clin Exp Allergy. 2006;36:1011‐1018. 16911357 10.1111/j.1365-2222.2006.02526.x

[3] Belgrave DC , Granell R , Simpson A , et al. Developmental profiles of eczema, wheeze, and rhinitis: two population‐based birth cohort studies. PLoS Med. 2014;11:e1001748. 25335105 10.1371/journal.pmed.1001748 PMC4204810

[4] Hill DA , Spergel JM . The atopic march: critical evidence and clinical relevance. Ann Allergy Asthma Immunol. 2018;120:131‐137. 29413336 10.1016/j.anai.2017.10.037 PMC5806141

[5] Hill DJ , Hosking CS , de Benedictis FM , Oranje AP , Diepgen TL , Bauchau V . Confirmation of the association between high levels of immunoglobulin E food sensitization and eczema in infancy: an international study. Clin Exp Allergy. 2008;38:161‐168. 18028467 10.1111/j.1365-2222.2007.02861.x

[6] Venkataraman D , Soto‐Ramírez N , Kurukulaaratchy RJ , et al. Filaggrin loss‐of‐function mutations are associated with food allergy in childhood and adolescence. J Allergy Clin Immunol. 2014;134:876‐82.e4. 25174864 10.1016/j.jaci.2014.07.033 PMC4186905

[7] Tsakok T , Marrs T , Mohsin M , et al. Does atopic dermatitis cause food allergy? A systematic review. J Allergy Clin Immunol. 2016;137:1071‐1078. 26897122 10.1016/j.jaci.2015.10.049

[8] Tsakok T , Woolf R , Smith CH , Weidinger S , Flohr C . Atopic dermatitis: the skin barrier and beyond. Br J Dermatol. 2019;180:464‐474. 29969827 10.1111/bjd.16934

[9] Brough HA , Nadeau KC , Sindher SB , et al. Epicutaneous sensitization in the development of food allergy: what is the evidence and how can this be prevented? Allergy. 2020;75:2185‐2205. 32249942 10.1111/all.14304 PMC7494573

